# Bringing ecology blogging into the scientific fold: measuring reach and impact of science community blogs

**DOI:** 10.1098/rsos.170957

**Published:** 2017-10-04

**Authors:** Manu E. Saunders, Meghan A. Duffy, Stephen B. Heard, Margaret Kosmala, Simon R. Leather, Terrence P. McGlynn, Jeff Ollerton, Amy L. Parachnowitsch

**Affiliations:** 1 UNE Business School/School of Environmental and Rural Sciences, University of New England, Armidale, New South Wales 2351, Australia; 2 Department of Ecology and Evolutionary Biology, University of Michigan, Ann Arbor, MI 48109, USA; 3 Department of Biology, University of New Brunswick, Fredericton, New Brunswick, Canada E3B 5A3; 4 Department of Organismic and Evolutionary Biology, Harvard University, Cambridge, MA 02138, USA; 5 Crop and Environment Sciences, Harper Adams University, Edgmond, Newport TF10 8NB, UK; 6 Department of Biology, California State University Dominguez Hills, Carson, CA 90747, USA; 7 Department of Entomology, Natural History Museum of Los Angeles County, Los Angeles, CA 90007, USA; 8 Faculty of Arts, Science and Technology, University of Northampton, Avenue Campus, Northampton NN2 6JD, UK; 9 Department of Plant Ecology and Evolution, Evolutionary Biology Centre, Uppsala University, Norbyvägen 18D, Uppsala 75236, Sweden

**Keywords:** communication, science community, blogging, impact

## Abstract

The popularity of science blogging has increased in recent years, but the number of academic scientists who maintain regular blogs is limited. The role and impact of science communication blogs aimed at general audiences is often discussed, but the value of science community blogs aimed at the academic community has largely been overlooked. Here, we focus on our own experiences as bloggers to argue that science community blogs are valuable to the academic community. We use data from our own blogs (*n* = 7) to illustrate some of the factors influencing reach and impact of science community blogs. We then discuss the value of blogs as a standalone medium, where rapid communication of scholarly ideas, opinions and short observational notes can enhance scientific discourse, and discussion of personal experiences can provide indirect mentorship for junior researchers and scientists from underrepresented groups. Finally, we argue that science community blogs can be treated as a primary source and provide some key points to consider when citing blogs in peer-reviewed literature.

## 1. Introduction

Scientific blogging has become increasingly popular over the past decade, but is still only undertaken by a small proportion of practising scientists. This may partly reflect uncertainty about what blogs are for and how time-investment in blogging can return benefits to scientific careers and to science generally. In this article, we provide a rationale for blogging in the field of ecology (very broadly defined to include aspects of evolutionary biology, conservation, systematics and environmental science). While there is a burgeoning academic literature on science blogging from a communication perspective, we believe this is the first time practising scientists who blog have analysed their own experience. We focus in particular on what we call science community blogging, as opposed to the more common and better-studied science communication blogging. Briefly, ‘science communication blogs' disseminate scientific information with their main target audience being non-specialists and the general public. ‘Science community’ blogs, in contrast, focus on issues about the process and culture of science and academia, with their main target audience being other scientists. There is, of course, overlap between these types of blogging and we return to the distinction below.

We ask two questions about the roles and value of science community blogs. First, how do we quantify blog audience and reach and what factors influence that reach? Second, what value does blog content hold for the community of working scientists? We then discuss the value of science community blogs to the academic community, as well as their role as a primary source. Our perspective is that of active science community bloggers (table 1) and blog readers. We believe that engaging with blogs, either as author or reader, can make significant contributions to ecology and other fields. We believe that there is an important niche for science community blogging, despite the fact that science community blogs are understudied or even ignored in many treatments of blogging. For example, science community blogs are not mentioned in Wilcox *et al*.'s [1] book *Science blogging: the essential guide*, which aims to provide a comprehensive contemporary analysis of the practice and impact of science blogs [2]. Science community blogs are also not explicitly represented in most scholarly treatments of science blogs. While some authors note the value of science blogs to the academic community, e.g. as a discussion or peer review forum, most studies focus on the role of science blogs as public education platforms and the distinction between science community and science communication blogs is not widely acknowledged (e.g. [3–7]).

**Table 1.** Details of the authors' blogs, including abbreviations used elsewhere in this article.

Blog title	abbreviation	Author(s)
Dynamic Ecology	DE	Duffy^a^
Small Pond Science	SPS	McGlynn, Parachnowitsch^a^
Scientist Sees Squirrel	SSS	Heard
Ecology Bits	EB	Kosmala
Jeff Ollerton's Biodiversity Blog	JOBB	Ollerton
Don't Forget the Roundabouts	DFR	Leather
Ecology is Not a Dirty Word	ENDW	Saunders

^a^These blogs have additional contributors who did not co-author this paper. DE: Jeremy Fox, Brian McGill; SPS: Catherine Scott.

## 2. What are blogs for?

Academics value a broad range of scholarly activities, including research, teaching, academic service and outreach. Among these, the outreach component of the academic portfolio is gaining increasing attention. Outreach can be defined in a variety of ways, but most broadly it means communication (including teaching and training) beyond the obvious audiences of (i) undergraduate and graduate students in our own degree programmes and (ii) academic peers in our own sub-disciplines. Outreach can target both scientific audiences (which have science training, but include non-specialists in the communicator's research area) and general ones (which have little or no formal science training). Outreach is a required part of life for many academics. Many research funding applications in the USA, UK and Australia must contain a statement about pathways to broader impacts; research assessment exercises such as the UK's Research Excellence Framework (REF) include measures of outreach (and subsequent societal impact), and some university contracts specify outreach as part of the job (e.g. Leather's university requires an annual report of outreach from each academic). The heavy emphasis on journal impact and research income as metrics of career advancement, however, means that outreach is often only paid lip service.

Perhaps one of the easiest ways for a scientist to reach a wider audience quickly is by blogging [8–10]. Blogging has evolved since its origin as an online personal journaling and bookmarking tool, to become a popular way to publish comment and opinion [11,12]. Many active and popular blogs written by scientists are science communication blogs with extremely broad followings. Other blogs are science community blogs, intended primarily to be read by other scientists. These often address the culture and process of science, offer advice to early-career scientists, discuss academic writing and publishing, consider issues of methodology and so on. There is also overlap between the science communication and science community categories, with some blogs addressing both audiences with a mix of post topics and writing styles.

Although science blogging (of both types) has become more established in recent years [13], few scientists have attempted to evaluate either the impacts of blogs or the benefits to bloggers [9]. This is particularly true for science community blogs, because the (limited) literature on blogging has emphasized science communication blogs. Our blogs (table 1) are predominantly science community blogs, although several also include some proportion of science communication content. Here, we focus on science community blogging, as we believe it deserves more attention as an important medium with goals and impacts that are distinct from those of science communication blogs.

Measuring the impact of scholarly activities is important to many academics, for a variety of reasons. First, academics wish to understand whether particular activities influence various audiences; for many, inspiring future knowledge generation is a key goal. Second, evidence of reach or impact beyond peer-reviewed literature can also be a requirement for funding or employment applications (e.g. the US National Science Foundation's Broader Impacts criterion).

Here, we discuss the potential reach, impact and value of science community blogging. In research, citation data for individual papers and h-index calculations for researchers are two of the most obvious metrics of academic impact. While these metrics have their shortcomings [14], they are more informative than simple paper-counting. By contrast, methods for quantifying reach, impact and value of blogs are not as well developed and are usually limited to the readership statistics available to individual blog authors through their blogging platforms. We draw on available statistics from our own blogs with the aim of stimulating more extensive discussion and analysis of the value of science community blogging.

## 3. Factors influencing reach and audience

To address our first question on how to identify the reach of science community blogs, and what factors influence that reach, we analysed data from our own blogs (table 1). All blog data were downloaded between 7 and 11 June 2017, covering the period from the blog's launch through May 2017 (table 2). Blog views and interactions (i.e. reach) change daily and a blog's overall reach usually increases with time, so these data represent the reach of each blog at the time of data collection.

**Table 2.** Summary data for each blog. See table 1 for blog abbreviations and electronic supplementary material for raw data.

blog	start date	average posts/ month	total followers	total comments	total visitors	total views	top views by country of origin (%)	top views by referrer (%)
DE	June 2012	21	9248	21 265	1 178 092	2 587 900	USA (50%)	search engines (24%)
SPS	Feb 2013	11	2160	4354	572 243	1 000 766	USA (60%)	search engines (29%)
SSS	Jan 2015	7	2873	2336	124 858	215 148	USA (41%)	Twitter (21%)
EB	Jan 2016	3	54	261	46 115	59 632	USA (56%)	Facebook (27%)
JOBB	Mar 2012	5	550	2074	79 052	123 936	UK (41%)	Facebook (24%)
DFR	Jan 2013	3	259	865	68 072	119 038	UK (37%)	search engines (48%)
ENDW	Oct 2009	2	473	859	28 290	53 479	Australia (31%)	search engines (20%)

The blogs vary significantly in age: the oldest blog started in October 2009 and the youngest in January 2016 (table 2). In the case of Dynamic Ecology (DE), we defined blog commencement as June 2012, because posts prior to this were an extension of editorial content from the *Oikos* journal written predominantly by DE founder (and former *Oikos* associate editor) Jeremy Fox. Fox's association with the *Oikos* blog ended in 2012 and the first post of the current blog ‘Dynamic Ecology’ was published in June 2012 [15]. For each blog, we collected data on: (i) followers, page views and posting frequency since blog commencement and (ii) page views and audience interactions for the top 10 most-viewed posts from each blog. We use the top 10 posts for each blog as a proxy to investigate how audience interactions may influence the reach of an individual blog post. All statistical tests presented were conducted in PAST v. 3.10 [16]. All correlation statistics are Spearman's (*r*_s_) correlations and assessments of significance are unchanged by sequential Bonferroni correction within sets of correlates (not shown). There are many confounding factors that influence the ‘success’ of a blog so it is hard to draw concrete parallels on what makes a popular post or blog. We highlight some of the challenges involved in quantifying blog reach and potential impact. A major caveat of our analysis is that we all use the WordPress blogging platform, and so our data are limited by the statistics available to authors on this platform. We therefore focus on general metrics that we believe are also relevant to most other platforms.

### 3.1. Measuring the reach of individual blog sites

Perhaps unsurprisingly, there is a strong relationship between posting frequency and many measures of blog traffic and audience interaction. The number of posts published on a blog per month was strongly correlated with its total number of registered followers (*r*_s_ = 0.86, *p* = 0.01), total comments on blog posts (*r*_s_ = 0.96, *p* < 0.001), total visitors (*r*_s_ = 1, *p* < 0.001) and total page views (*r*_s_ = 1, *p* < 0.001). However, blog longevity (the number of months the blog has been publishing online) had no influence on the total number of followers (*r*_s_ = 0.14, *p* = 0.71), total number of comments (*r*_s_ = 0.07, *p* = 0.84) or total visitors (*r*_s_ = −0.14, *p* = 0.71).

**Figure 1.**
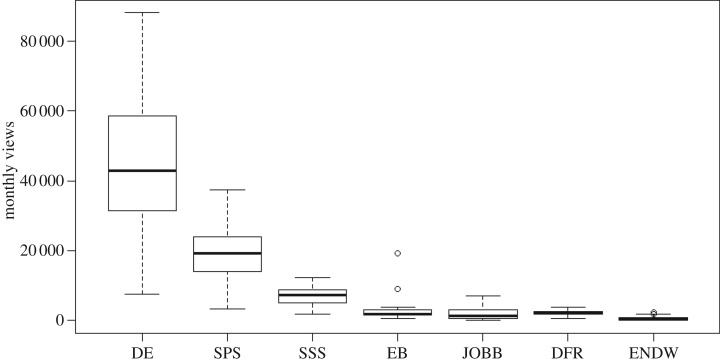
Median monthly blog views for the entire sample period for each of the seven blogs represented in this analysis. See table 1 for blog codes.

Measuring the reach of science community blogs is difficult, because individual blogs differ widely in their content and audience interactions. We found that median monthly blog views differed significantly among individual blogs (Kruskal–Wallis test: *H_c_* = 306.5, *p* < 0.001; figure 1). In particular, the two multi-author blogs (DE, SPS) have greater readership than the single-author blogs. This seems intuitive, because each additional author adds another personal network of potential readers, but our sample is too small to confirm this trend. North American blogs (DE, SPS, SSS, EB) also received higher average monthly views than UK and Australian blogs (JOBB, DFR, ENDW), which may reflect the stronger blog culture in North America [17], or the time-zone effect inherent in global social media networks [18]. We also asked whether readership followed seasonal patterns, where holiday seasons may receive fewer monthly views; however, a two-way ANOVA using our blog data showed that blog identity (*F*_6,282_ = 23.01, *p* < 0.001) influenced monthly views more than the calendar month (*F*_11,282_ = 0.97, *p* = 0.47) and there was no interaction between the two main effects (*F*_66,282_ = 1.02, *p* = 0.45).

**Figure 2.**
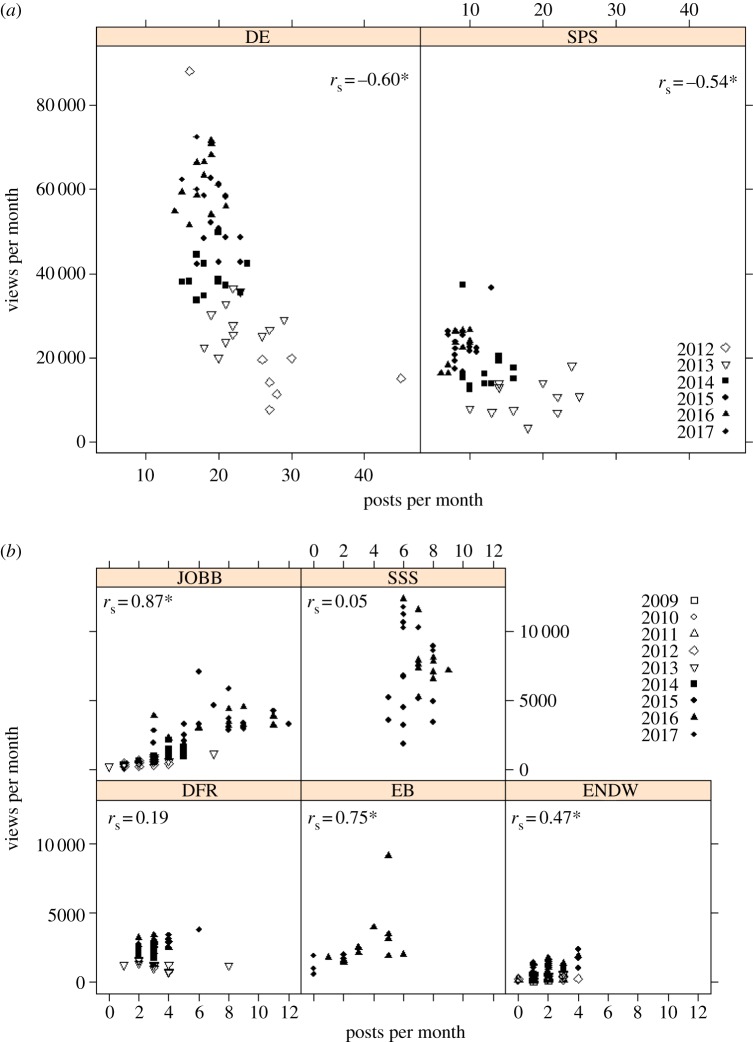
Relationship between monthly views and number of posts published per month for (*a*) group author blogs and (*b*) single-author blogs. Asterisks next to correlation coefficients indicate *p* < 0.001. One extreme outlier was removed from EB to improve readability. See table 1 for blog abbreviations and electronic supplementary material for raw data.

The strength and direction of the correlation among monthly posting frequency and monthly views varied between individual blogs (figure 2). This suggests that higher posting frequency is not, on its own, an indicator of audience engagement. The two group blogs (DE, SPS) showed a strong negative correlation between monthly views and posting frequency (figure 2*a*), while the single-author blogs showed positive or no correlation between monthly posts and views (figure 2*b*). The two group blogs have a considerably higher average posting frequency compared with the other blogs (table 2); however, these two blogs posted more frequently in the first 1–2 years and have reduced frequency over time, while posting frequency on the single-author blogs has increased or remained stable as readership increased. The exception is EB, the youngest of the blogs, which shows a strong positive relationship between posts and views despite the author (Kosmala) not publishing any posts since February 2017. However, the positive relationship may be partly driven by a couple of posts that became ‘viral’, including EB's most viewed post of all time [19] that received over 24 500 more views than the average views for her top 10 posts and was shared over 5000 times on Facebook (see the next section for discussion of individual posts). These results suggest that overall reach of a science community blog is influenced by a complex relationship between posting frequency, blog profile (including author identity), and the impact of individual posts.

### 3.2. Measuring the reach of individual posts

Prior studies have shown that most readers visit original blogs predominantly for opinions and analysis [20,21]. In particular, Jarreau & Porter [13] identified ‘the author's perspective’ as one of the top motivators for science blog readers. Therefore, measuring content of posts, i.e. quality, rather than just the number of posts, may be more useful for those seeking to measure blog impact. However, explicitly quantifying the reach and impact of an individual blog post is an elusive goal. The average number of views for the top 10 posts on each blog was not correlated with the number of blog followers (*r*_s_ = 0.64, *p* = 0.11), indicating that popular posts have substantial reach beyond the blog's regular following. Page views for individual top 10 posts were positively correlated with the number of times the post was shared on Facebook (*r*_s_ = 0.32, *p* < 0.01), the number of comments on the post (*r*_s_ = 0.52, *p* < 0.01) and the number of external links to the post from other blogs (called ‘pingbacks’; *r*_s_ = 0.55, *p* < 0.01). There are, however, some important caveats to consider if using these simplified metrics as evidence of reach (and potential impact), as they are not necessarily accurate estimates of true engagement.

First, individual posts attract readers from multiple sources. The ‘Facebook shares’ metric we use here is not representative of all social media shares, but this was the only measure of social media shares for individual posts available to us through our blogging platform. We were unable to gather data on Twitter shares for each post, as Twitter disabled that function in 2015 [22]. However, Twitter and Facebook were in the top three referrers (external sites from which readers arrive via a page link) of all time for all blogs except SSS, for which only Twitter was in the top three. Our data suggest that social media referrals extended the reach for each blog site overall, beyond the pool of subscribed followers: the proportion of total visitors to each blog that was referred from the combined social media (Twitter + Facebook) was negatively correlated with the total number of blog followers (*r*_s_ = −0.82, *p* = 0.03). Authors on other science community blogs can also influence the reach of another blog by linking to individual posts in their own content. For example, all our blogs except DE registered another science community blog in their top five referring sites of all time—three of these (SSS, SPS, JOBB) received a high number of links from DE, while EB, DFR and ENDW registered high referrals from other science community blogs beyond our sample. While these links can be measured via pingbacks, the listing of pingbacks on each post is not enabled on all sites and links made from mainstream news or non-blog websites are often not registered. We only counted external pingbacks here as a measure of external impact, but total number of pingbacks on a blog post also include links from later blogs by the same author. Depending on the goal of measurement, intra-site pingbacks can be treated similarly to self-citations in scholarly literature, which is an important part of building a body of work.

Second, the number of comments left by readers on a post also explains very little about that post's reach and potential impact, as this number includes replies by the blog author and comment conversations involving multiple back-and-forth replies between commenter and author. Additionally, not every visitor who reads the post will leave a comment, and we have found that many readers prefer to comment about the post on their own social media feeds (e.g. Twitter), rather than on the post itself. If comparing comments between blog sites, it is also important to remember that some authors may close comments on older posts, which restricts the engagement of readers who find the post in the future.

**Figure 3.**
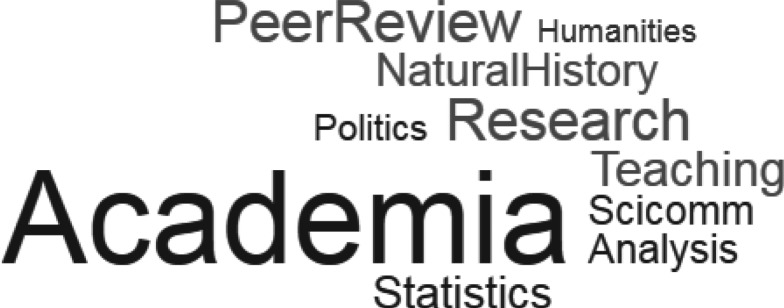
Word cloud created from the topics of all top 10 posts from our sample blogs (*n* = 70). Larger words indicate higher frequency of occurrence. Posts were assigned to general categories based on the content and opinions expressed in the post. ‘Academia’, broader academic issues and experiences relevant across multiple disciplines; ‘PeerReview’, posts about reviewing papers or the peer review system; ‘Research’, methods or issues relating to research practice; ‘Teaching’, posts directly relevant to teaching undergraduate students; ‘NaturalHistory’, posts on natural history or ecology of a species; ‘Scicomm’, posts focused on fact-checking media coverage or communicating a topical scientific issue to non-specialist audiences; ‘Analysis’, posts providing analysis of published research; ‘Statistics’, posts about statistical issues or components; ‘Politics’, posts focused on topical political issues; ‘Humanities’, posts comparing humanities and science disciplines. See the electronic supplementary material for data.

Third, quantification metrics explain little about the content of the post, which is a key driver of the reach and potential impact of individual posts. The most common topics covered by our top 10 blog posts (figure 3) suggest that expert advice or opinions on broadly relevant academic and teaching issues attract a larger readership (e.g. [23]), which matches the broader motivations of blog readers generally [13,20].

Finally, reach is not synonymous with impact. We are unable to quantify impact with our data (in fact, ‘impact’ or ‘participation’ from communication activities is notoriously hard to quantify [24,25]). From our own experiences, we have felt impact from our blogs on a more personal level than the readership stats provided here. As some examples: Saunders' most viewed post of all time is ‘On the importance of observations to ecology’ [26] and a number of colleagues who did not engage with the post online commented to her in person that they were inspired to read a post strongly supporting the value of field observations for an ecology research career; Duffy notes a woman approaching her in a local park to tell her that she lost a lot of guilt after reading Duffy's post on not needing to work an 80-hour-week [27], as she thought she would not get tenure because she did not work that long; and several early career scientists have told Heard that his post on how introverts can handle conferences [28] has helped them attend, and network at, professional conferences.

In summary, it is difficult to compare blog sites in terms of reach and impact, due to large variability in authorship, writing style, content focus and geographical networks. For individual bloggers trying to quantify their reach and impact, we recommend that authors use a range of metrics and analyses, rather than just simplified values like ‘number of followers’. For authors trying to expand their reach, we have found that engaging with other social media platforms beyond the blog itself (particularly Twitter and other science community blogs) significantly increases blog reach, and this relationship has been noted by other researchers [6].

## 4. Value of science community blogging to the scientific community

What value does blog content hold for the community of working scientists? To answer this question, we discuss potential benefits of science community blogging using our own blogs and experiences as qualitative data. From an individual perspective, a key benefit of participating in science community blogging is the opportunity for career exposure and networking with national and international colleagues. Sustaining these networks builds a sense of community with peers, as well as an opportunity for early career scientists to interact with a broader community than they would otherwise have an opportunity to meet. We believe this is an important aspect of science community blogs that distinguishes them from science communication blogs. The benefits of such social connections have been discussed widely in the literature (e.g. [3,6,8]) and are equally relevant for readers who comment and participate in discussions on blogs, or interact with blogs via other social media (e.g. Twitter). While these interactions certainly can benefit individuals, we argue that they also benefit the scientific community by facilitating the exchange of ideas (and this is reflected in the inclusion of blog citations of papers within Altmetrics scores). Indeed, science community blogging can have tangible benefits in its ability to foster academic collaborations. Batts *et al*. [3] discuss how blogs can influence academia by providing a fast-turnaround forum for peer review, using the example of Comai & Cartwright's [29] collaborative criticism of a published paper that arose from an original critical blog post by Cartwright. Blog network interactions can also create new collaborations that may otherwise not eventuate. For example, our paper came about because of long-term interactions with each other's blogs, and was inspired by a post on DFR about blog readership data [30]. Some of us have also found external collaborations through blog posts. For example, a recent guest post on DE by Ollerton and Angela Moles [31], who had never previously collaborated, was turned into a peer reviewed opinion paper in *Biotropica* at the invitation of that journal's editor [32]. An interaction on Twitter between Leather and another ecologist resulted in a joint blog post [33], which subsequently led to an edited version of the post being published in popular ecology magazine *BioSphere*. Similarly, a recent blog post on DFR resulted in an invitation to produce a peer-reviewed opinion piece [34], and a post on SSS has sparked a (current) research collaboration among scientists at four institutions, none of whom had previously met. Therefore, we suggest that science community blogging can have broad benefits in developing the academic community in terms of professional networks, collaborations and the literature, and these benefits can also represent impact for the blog author.

Regular science blogging is an excellent method for improving writing and communication skills, and this individual benefit can spill over into broader benefits for the scientific community. Fostering the habit of writing regularly benefits scientists who may view ‘writing up’ the results of a research project as a chore. In particular, blog writing can be one way to deal effectively with the blank page syndrome that many academics struggle with, mainly because writing for a blog need not follow scientific and publishing conventions. As some of us have experienced, writing a blog post (or other less formal piece) can help overcome writer's block on an academic paper. Broader adoption of the blogging habit could, as a result, benefit the scientific community by encouraging more scientists to disseminate their work and reduce the ‘file drawer’ problem [35] of data that never gets shared.

The valuable role of blogs as sources of indirect advice and mentorship is an often overlooked service to the scientific community. Blogs can be especially valuable for students, early career researchers, women and other minority groups, who are struggling with particular academic or personal issues but are unsure of who to ask for advice. These benefits also extend to broadening the conversation and promotion of diversity in academia. For example, McGlynn has used his blog to improve equity and opportunity for students at minority-serving institutions [23]. Pikas [36] found high centrality (a measure of connectedness) in blogs authored or co-authored by women and suggests that science blog communities may be an important source of mentorship and advice for women in academia. Our examples of ‘diffuse-mentorship’ posts range from thoughts on work/life balance in academia, mental illness, parenthood, women in science, introversion, early career researcher unemployment struggles and more (e.g. [28,37–40]). Other examples beyond our blog network exist (e.g. [41–43]). The blogging medium is valuable because it allows for sharing personal stories and experiences that can provide support ranging from strategies to deal with a particular situation to simple confirmation that the reader is not alone.

Finally, science community blogging provides a medium for content that is unlikely to find its way into the academic literature. We argue that this characteristic creates great potential for science community blogs to contribute to scientific discourse. For example, science community blogging can provide a venue for rapid communication of advances in teaching and research methods that circumvent the protracted process of writing and updating new textbooks. Three of Leather's top 10 posts were from his ‘Entomological Classics’ series, which cover key methods and equipment used in entomological field sampling. While there are a number of peer-reviewed journals focused on in-depth treatments of teaching ideas in ecology, blogging is an efficient way to share current and innovative ideas and applications that are broadly relevant to ecology, and to seek feedback on them (e.g. [44,45]). Blogging can also provide rapid communication of short notes (e.g. natural history observations, failed experiments or newly developed R code) or opinions that are useful to relevant peers, but would probably not be accepted for publication in most peer-reviewed journals (e.g. [46,47]). Science community blogs help to share these ideas with a wider audience beyond the annual conference water-cooler chat.

## 5. Incorporating science community blogs into the scientific literature

As a standalone medium, blogs provide rapid communication of scholarly ideas and opinions, a potentially broader discussion forum than journal papers that are often pay-walled, and remove barriers for a greater number and diversity of perspectives to join the conversation. They also provide a valuable medium for post-publication peer review and analysis, as well as observations, ideas or analyses for which there are limited examples available in the published literature. Therefore, we argue that science community blogs are an often overlooked primary source that can provide valuable citations for enhancing the discourse published in peer-reviewed literature. Blog posts are not a replacement for peer-reviewed literature and should not be seen as a way to circumvent that process. They are, however, similar to preprints and ‘personal communications’, which are often accepted as citations, so why not blogs? In fact, blogs have more value than personal communications, as the original content and context can be verified by the reader. Below, we discuss some key issues for researchers and editors to consider when deciding whether to incorporate science community blogs into the scientific literature.

### 5.1. Assessing credibility

Assessing credibility is not a simple task, because perceived credibility is largely driven by individual readers' beliefs and motivations [21,48]. Gender biases also influence people's perceptions, whereby female bloggers may be considered less credible than male bloggers [49]. As happens in the scholarly literature, the perceived credibility of a blog is also probably influenced by the author's position and reputation within their discipline. The identity of an author may influence their perceived credibility, but anonymity should not necessarily preclude a blog from being citable. In fact, Chesney & Su [50] found that blog presentation (formatting, proofreading) affected readers' perceived credibility, while anonymity of the author did not. We suggest that assessing credibility of science community blogs should be a similar process to assessing the credibility of the peer-reviewed literature. Observations and personal experiences can be taken at face value, especially (for example, in the case of natural history observations) if accompanied by photographic evidence, while arguments and in-depth analyses can be assessed on fairness, accuracy, links to sources and presentation.

### 5.2. Citing blogs in manuscripts

Although it is ultimately up to the reader to assign weight to any citation, it may be particularly useful for journals to encourage peer reviewers to check blog posts cited in manuscripts for relevance and credibility, especially if high numbers of blogs are cited relative to other forms of literature. We do not, however, advocate this as a gatekeeping exercise. An additional concern with citing blogs is their longevity compared to scholarly literature. An author can delete blog posts, or entire blog sites, with the click of a mouse, thereby bringing into question the validity of the citation for future readers. However, current citation practices for web pages include an access date to address the possibility of future changes. If authors citing blog pages have further concerns, archiving tools (e.g. Wayback Machine, https://archive.org/web/) may be used to provide stable URLs for future reference.

## 6. Conclusion

We hope we have convinced the reader of the value of science community blogging, both for the careers of individual scientists and for the science community in general. Scientific blogs are still a relatively new phenomenon in comparison with more traditional forms of communication, and discussions of their role in science are part of a broader debate about the future of scientific publishing. It is, however, worth reflecting that much of what we take for granted as twenty-first century scientists does not have a long history: peer review, currently the ‘gold standard’ of publication, was resisted by some journals even as late as the mid-1970s, while electronic submission of manuscripts and online publication is less than 20 years old. We believe science blogs are here to stay, and that science community blogs can play important roles in advancing scientific ideas and mentoring the next generation of scientists.

## Data accessibility

The data used for analysis have been deposited in Dryad (http://dx.doi.org/10.5061/dryad.kf8b0) [51].
